# Perry Syndrome with Intrafamilial Heterogeneity in Presentation and Survival Including Acute Respiratory Failure: Case Series

**DOI:** 10.1002/mdc3.13473

**Published:** 2022-06-01

**Authors:** Jeremy Boardman, Maria Mascareno Ponte, Amina Chaouch, Christopher Kobylecki

**Affiliations:** ^1^ Department of Neurology Lancashire Teaching Hospitals National Health Service (NHS) Trust Preston United Kingdom; ^2^ Department of Respiratory Medicine Manchester University NHS Foundation Trust Manchester United Kingdom; ^3^ Manchester Centre for Clinical Neurosciences, Northern Care Alliance NHS Foundation Trust, Manchester Academic Health Science Centre University of Manchester Manchester United Kingdom

**Keywords:** Perry syndrome, parkinsonism, respiratory failure, noninvasive ventilation, genetics

## Abstract

**Background:**

Perry syndrome is a rare autosomal dominant parkinsonian disorder characterized by respiratory failure. The variability in respiratory presentation in this condition is incompletely understood.

**Cases:**

We report 2 first‐degree relatives with Perry syndrome attributed to the same mutation in the Dynactin 1 *(DCTN1)* gene. Their clinical presentations with respect to parkinsonism and respiratory failure were heterogeneous. The proband presented with acute respiratory failure requiring invasive ventilation on a background of parkinsonism and remains alive more than 3 years later with a good levodopa response. We contrast this with the published literature, in which acute respiratory presentations were associated with a poor outcome. The proband's brother presented with parkinsonism together with early falls and gait impairment and died following gradual hypoventilation despite noninvasive respiratory support.

**Conclusions:**

Perry syndrome can show intrafamily heterogeneity in both movement disorder and respiratory presentations. Acute respiratory failure is often but not always associated with a poor outcome.

Perry syndrome was first described in 1975 by Perry et al following the study of 3 successive generations of a Canadian family.^1^ The literature describes a parkinsonian syndrome associated with depression, apathy, weight loss, and respiratory disturbance.^2^ It is inherited in an autosomal dominant fashion as a result of mutations on exon 2 of the Dynactin 1 *(DCTN1)* gene.^3^ We report 2 related cases of Perry syndrome showing intrafamilial heterogeneity in clinical presentation and the course of respiratory involvement, with 1 family member presenting in acute respiratory failure.

## Case 1

A 55‐year‐old woman was admitted to her local hospital with a 3‐week history of severe fatigue with diarrhea and vomiting. In the emergency department, she had multiple apneic episodes requiring intubation and ventilation. There was no obvious infective or metabolic derangement to account for her presentation.

She had been diagnosed with benign essential tremor 2 years previously due to a 7‐month history of bilateral action tremor of both upper and lower limbs. She was taking propranolol for this but no other prescribed or over‐the‐counter medications. In the intervening 2 years, she had lost 12 kg in weight and had been diagnosed with depression after becoming withdrawn and apathetic. There was said to be a family history of Parkinson's disease and multiple sclerosis, but more details were not initially available.

Her intensive care unit (ICU) admission was complicated by recurrent apneic episodes causing difficulty weaning, with worsening tremor. Due to jerky limb movements and a depressed conscious level, she was initially treated with levetiracetam and phenytoin. Apart from mild reversible hyponatremia, routine blood tests returned normal. She was transferred to the ICU at the regional neurosciences center.

Brain magnetic resonance imaging (MRI) was normal, and lumbar puncture revealed normal cerebrospinal fluid constituents (white cell count <1, protein 0.17 g/L, glucose 5.4 mmol/L with paired plasma glucose 8.8 mmol/L, and negative oligoclonal bands). Electroencephalogram showed encephalopathic features with continuous slow wave activity over the left frontotemporal region. Autoimmune encephalitis was suspected, and given the prodrome of weight loss and subacute diarrhea, we considered anti‐DPPX antibody syndrome. As such, she was treated with 3 days of intravenous methylprednisolone followed by high‐dose oral prednisolone.

Following respiratory wean, she was stepped down to ward‐level care. The encephalopathy and apneic episodes completely resolved during her inpatient stay. There was a striking improvement in mood compared with the withdrawn and apathetic affect of the previous 2 years. Examination revealed slight hypomimia and normal vertical gaze. She had a rest tremor of the right upper and lower limb with asymmetrical postural and action tremor worse on the right, and rest tremor of the tongue. There was mild right‐sided limb rigidity and moderate right upper limb bradykinesia. She commenced co‐careldopa, and at 100/25 mg three times daily (tds) there was a definite improvement in her tremor and bradykinesia. Overnight oximetry showed a desaturation index of 1.7/h, but she desaturated to below 85% for 12 minutes with a low of 65%, suggesting an element of sleep‐disordered breathing.

Immunology testing revealed negative anti‐glutamic acid decarboxylase, thyroid peroxidase, N‐methyl‐D‐aspartate receptor, Leucine Rich Glioma Inactivated‐1, Contactin‐associated‐protein‐like‐2, glycine receptor, α‐amino‐3‐hydroxy‐5‐methyl‐4‐isoxazolepropionic acid receptor 1 and 2, a, Dipeptidyl‐Peptidase–Like Protein‐6, and immunoglobulin‐like cell adhesion molecule 5 antibodies. Revisiting the family history, her mother had been diagnosed with idiopathic Parkinson's disease at 46 and died in her early 50s, whereas her 57‐year‐old brother (case 2) was being treated for rapidly progressive atypical parkinsonism with an erratic breathing pattern and had recently started on continuous positive airway pressure ventilation (CPAP).

Genetic testing revealed that the patient was heterozygous for the c.211G > A, p.(G71R) pathogenic mutation in exon 2 of the *DCTN1* gene, confirming a diagnosis of autosomal dominant Perry syndrome. When seen 4 months following discharge, she showed an excellent response to levodopa with no tremor, bradykinesia, or rigidity and mild levodopa‐induced dyskinesia on co‐careldopa 100/25 mg tds (Video 1). There were no symptoms of hypoventilation, and earlobe blood gas (ELBG) showed no evidence of hypoventilation partial pressure (p) CO_2_ 5.5 kPa, pO_2_ 12 kPa, serum bicarbonate (HCO_3_ 29 nmol/L). Spirometry was normal.

**Video 1:** Case 1 after commencing treatment with levodopa. Video taken approximately 90 minutes after co‐careldopa 100/25 mg dose. Mild levodopa‐induced dyskinesia with no significant bradykinesia or tremor.

After a further 6 months, ELBG showed pCO_2_ 6.3 kPa, pO_2_ 12.4 kPa, and HCO_3_ 30.1 nmol/L. When reviewed 12 months following discharge, ELBG confirmed a compensated type 2 respiratory failure (pH 7.40, pCO_2_ 6.6 kPa, pO_2_ 9.5 kPa, HCO_3_ 28.3 mmol/L). She did not disclose symptoms of hypoventilation. She was eventually commenced on noninvasive ventilation (NIV), although she did not tolerate this well. She remains alive at 3½ years following her initial presentation with respiratory failure, although weight loss continues to be a significant problem. She is currently taking co‐careldopa 100/25 mg four times daily (qds) with a sustained good motor response and mild dyskinesia. She is also taking sertraline 150 mg daily for depression.

## Case 2

Her brother presented at the age of 55 with impaired balance with early falls and gait freezing for 2 years. There was no history of tremor. There was no history of sleep disturbance or sleep‐disordered breathing. On examination, he had right greater than left‐sided bradykinesia and rigidity, with no evidence of tremor. He reported dysphagia for solids and liquids at presentation.

Brain MRI was normal, and [^123^I]‐Ioflupane single photon emission computed tomography  imaging showed bilateral dopamine denervation within the putamen.

He did not respond to treatment with co‐careldopa 100/25 mg 5 times daily and rotigotine 4 mg/24 h, and his balance problems and falls rapidly worsened. At 1 year from his original evaluation, he was noted to have erratic breathing during sleep and was initially diagnosed with sleep apnea. He was more withdrawn and showed a tendency to cram food into his mouth when eating. He had marked rigidity and bradykinesia in all 4 limbs (Video 2), with postural instability on the pull test. He required a wheelchair to mobilize. Genetic testing confirmed a p.(G71R) mutation in *DCTN1* gene.

He commenced respiratory support with CPAP but died 3 years after his initial evaluation.

**Video 2:** Case 2 showing significant symmetrical bradykinesia despite levodopa treatment (approximately 60 minutes post co‐careldopa 100/25 mg dose). Disordered breathing and inspiratory sighs are heard on the recording.

## Discussion

Since the original description of Perry syndrome in 1975, case reports and series have informed understanding of the different clinical presentations of this condition. In 2017, Mishima et al proposed diagnostic criteria for Perry syndrome.^4^ The cardinal features of these are parkinsonism, apathy or depression, respiratory symptoms, unexpected weight loss, and a family history of parkinsonism or respiratory difficulties. Our cases fulfill the criteria for “definite Perry syndrome” given that they had confirmed mutations in the *DCTN1* gene.

Case 1 is unusual due to the fulminant presentation of respiratory failure with no preceding respiratory symptoms. Initially, weaning from invasive ventilation was slow, but subsequently the recurrent apneic episodes resolved and clinically the patient was free from respiratory difficulties on discharge. Respiratory involvement is seen in 66% of reported patients with Perry syndrome but is thought to be a late complication of the condition.^2^, ^4^ We summarize reported cases of Perry syndrome with acute respiratory presentations in Table 1.^1^, ^5^, ^6^, ^7^, ^8^ Acute respiratory failure occurred at a symptom duration between 1 and 6 years, with all patients requiring mechanical ventilation and with tracheostomy in 5 cases. All patients who survived to discharge required long‐term ventilation with variable survival reported; 1 patient was treated with diaphragmatic pacing.^8^ Case 1 is therefore unusual due to the long latency to requiring NIV following presentation with acute respiratory failure and the relatively long survival to date. The mechanism of respiratory involvement in Perry syndrome is likely multifactorial, and the reason for prolonged respiratory stability following acute admission in our case is unclear. Depletion of medullary tyrosine‐hydroxylase and serotoninergic neurons has been linked to respiratory involvement in Perry syndrome.^9^ It is an intriguing possibility that dopaminergic treatment may have resulted in some improvement in her respiratory status as well as the mood and motor symptoms. Careful monitoring of people with Perry syndrome to allow the detection and treatment of respiratory complications is of critical importance in their management.

**TABLE 1:** Cases of Perry syndrome with acute respiratory presentations reported in the literature, including the proband in this case (case 1)

Study, Country	Sex	Prior Respiratory Symptoms	Acute Presentation	Disease Duration, Years	Mutation	Acute Respiratory Support Required	Ongoing Respiratory Support Required After Discharge
Perry et al, Canada, 1975^1^	Male	Difficulty in breathing unrelated to exertion	Respiratory arrest	5	p.G71R	Tracheostomy and mechanical ventilation	Died on ventilator
Elibol et al, Turkey, 2002^5^	Male	Respiration noticed to be more superficial	Severe respiratory failure and confusion	4	p.G71R	Mechanical ventilation	Tracheostomy and home ventilator for intermittent support particularly overnight; died 11 months postadmission
Newsway et al, United Kingdom, 2010^6^	Male	Sleep‐disordered breathing	Found unresponsive following respiratory arrest	6	p.G71R	Mechanical ventilation	Nocturnal NIPPV
Ohshima et al, Japan, 2010^7^	Female	None reported	Sudden lethargy and respiratory acidosis	3.5	p.Q74P	Mechanical ventilation	Tracheostomy, nocturnal NIPPV with occasional daytime ventilation; died aged 65 after >6 months
	Male	Ataxic breathing	Loss of consciousness and respiratory acidosis	1		Mechanical ventilation	NIPPV; died after 2 years
	Male	Subjective shortness of breath	Respiratory arrest without prior warning of respiratory inadequacy	3		Tracheostomy and mechanical ventilation	Nocturnal ventilation; died after 1 year
Pretelt et al, Colombia, 2014^8^	Female	None reported	Acute deterioration of respiration	2	p.G71R	Mechanical ventilation via tracheostomy	Fitted with bilateral diaphragmatic pacemaker due to failure to wean off ventilator; well at 2 years postventilation
Case 1	Female	None reported	Acute respiratory failure	2.5	p.G71R	Mechanical ventilation via tracheostomy (decannulated prior to discharge)	Nil initially; NIPPV started 18 months postadmission but poorly tolerated; remains alive 3.5 years postrespiratory failure

Abbreviation: NIPPV, noninvasive positive pressure ventilation.

Interestingly, all cases reported of acute respiratory failure in Perry syndrome, aside from the Japanese series, have the G71R mutation. No definite clinicogenetic correlations of respiratory involvement in Perry syndrome are yet established,^4^ and further data are needed to determine the risks for acute respiratory presentation.

Whereas case 1 showed excellent dopaminergic response, with some levodopa‐induced dyskinesia, levodopa response was poor in case 2, with rapid development of axial symptoms, including falls and dysphagia. We acknowledge that response of some symptoms to higher doses of levodopa has been seen in some reported cases,^10^ but in this case the mainly axial and gait‐related symptoms would not have been expected to show a good response to higher doses. Respiratory involvement was more insidious, with minimal response to respiratory support. Clinical heterogeneity has been reported in families with Perry syndrome, with variations in levodopa response and the development of respiratory symptoms.^2^, ^4^ The variation in both parkinsonian phenotype and respiratory course in our family exemplifies this. Brain MRI was normal in both cases, as is often the case in the literature.^10^

We acknowledge limitations to our work. Autopsy was not performed in case 2, although the patient has “definite Perry syndrome” according to current diagnostic criteria due to the confirmed *DCTN1* mutation. We report only 2 patients from our family as more information on the mother who had presumed Perry syndrome was not available.

Our report highlights the clinical heterogeneity in a new family with definite Perry syndrome. In addition, we provide evidence in 1 case of a long period of stability without respiratory support following admission with acute respiratory failure, not to our knowledge previously documented in this condition. As more cases of this condition are reported, our understanding of the spectrum of respiratory complications of Perry syndrome and survival postrespiratory failure will continue to expand.

## Author Roles

(1) Research Project: A. Conception, B. Organization, C. Execution; (2) Manuscript: A. Writing of the First Draft, B. Review and Critique.J.B.: 1A, 1B, 1C, 2A

M.M.P.: 1B, 1C, 2B

A.C.: 1B, 2B

C.K.: 1A, 1B, 1C, 2A, 2B

## Disclosures

**Ethical Compliance Statement:** The authors confirm that the approval of an institutional review board was not required for this work. Written informed consent has been obtained to publish these cases and to publish video material. We confirm that we have read the Journal's position on issues involved in ethical publication and affirm that this work is consistent with those guidelines.

**Funding Sources and Conflicts of Interest:**No specific funding was received for this work.

**Financial Disclosures for the Previous 12 Months:** J.B., M.M.P., and A.C. report no specific disclosures. C.K. has received grants from Parkinson's UK and The Michael J. Fox Foundation and speaker fees from Britannia and Bial Pharma.
